# Improved Ophthalmic Outcomes Following Venous Sinus Stenting in Idiopathic Intracranial Hypertension

**DOI:** 10.3389/fopht.2022.910524

**Published:** 2022-06-30

**Authors:** Kafayat A. Oyemade, Timothy T. Xu, Waleed Brinjikji, Jeremy K. Cutsforth-Gregory, Giuseppe Lanzino, David F. Kallmes, Heather E. Moss, Robert Dodd, M. Tariq Bhatti, John J. Chen

**Affiliations:** ^1^ Alix School of Medicine, Mayo Clinic, Rochester, MN, United States; ^2^ Department of Ophthalmology, Mayo Clinic, Rochester, MN, United States; ^3^ Department of Radiology, Mayo Clinic, Rochester, MN, United States; ^4^ Department of Neurologic Surgery, Mayo Clinic, Rochester, MN, United States; ^5^ Department of Neurology, Mayo Clinic, Rochester, MN, United States; ^6^ Department of Neurology and Neurological Sciences, Stanford University, Palo Alto, CA, United States; ^7^ Department of Ophthalmology, Stanford University, Palo Alto, CA, United States; ^8^ Department of Neurosurgery, Stanford University, Palo Alto, CA, United States

**Keywords:** idiopathic intracranial hypertension, pseudotumor cerebri, chronic intracranial venous hypertension syndrome, venous sinus stenting, endovascular

## Abstract

**Background:**

Ophthalmic outcomes following venous sinus stenting (VSS) in patients with idiopathic intracranial hypertension (IIH) are not well characterized.

**Materials and Methods:**

A retrospective chart review was conducted on 86 consecutive patients with IIH who underwent venous sinus stenting at Mayo Clinic, Rochester, Minnesota and Stanford Medical Center, Palo Alto, California between May 2015 and October 2021. Patients with raised intracranial pressure from a non-IIH etiology were excluded. Clinical symptoms and neuro-ophthalmological data, including best corrected visual acuity (BCVA), visual field mean deviation, papilledema, and optical coherence tomography (OCT) peripapillary retinal nerve fiber layer (pRNFL) and ganglion cell inner plexiform layer (GC-IPL), were analyzed. Baseline measurements before VSS and 3 months or more postoperatively were compared.

**Results:**

Eighty-six subjects (82 female) were included in this study, with a median age of 33 (16–68) years and a median body mass index of 36.69 (22.30–62.00) kg/m^2^. 85/86 (98.8%) had attempted prior management with medication, and 12/86 (14%) had prior surgical management with optic nerve sheath fenestration, ventriculoperitoneal shunt, or bariatric surgery. Prior to VSS, 67/86 (77.9%) had papilledema, 85/86 (98.8%) had headaches, and 68/86 (79.1%) had pulsatile tinnitus. For patients with both pre- and post-VSS data available, the average papilledema grade was 1.76 (0–5) (n = 74) and the average OCT pRNFL was 186.34 (52.00–588.00) µm (n = 70), prior to VSS. A median of 4.0 (interquartile range 3.3–5.4) months after VSS, the average papilledema grade improved to 0.39 (0–2), *p* <0.001, and OCT pRNFL improved to 96.86 (47.00–168.00) µm, *p* <0.001. 28/86 (32.6%) patients no longer required medication for high intracranial pressure. 14/85 (16.5%) patients reported complete resolution of their headache and 55/71 (77.5%) reported improvement in headache quality. 40/68 (66.7%) reported complete resolution of pulsatile tinnitus. OCT GC-IPL, BCVA, and visual field mean deviation did not significantly change pre- vs post-VSS.

**Conclusions:**

Our large consecutive case series corroborates smaller prior studies in demonstrating the overall efficacy of VSS for patients with IIH. We found both ophthalmic improvements, as demonstrated by the significantly reduced papilledema and pRNFL, and overall clinical symptom improvement.

## Introduction

Idiopathic intracranial hypertension (IIH) is characterized by symptoms and signs of elevated intracranial pressure (ICP) without a known cause. IIH is the most common cause of raised ICP and papilledema and occurs in 1–2.4 per 100,000 people each year (1–4). It predominantly affects obese women of child-bearing age, showing an almost twenty-fold increase in incidence in this segment of the population (19–21 per 100,000) and increasing in incidence along with the worldwide obesity epidemic (1, 2, 4). Although it was previously called benign intracranial hypertension, IIH can cause significant vision loss (5, 6). The clinical presentation of IIH can be quite variable but is typically characterized by headaches, transient or permanent visual changes, diplopia, nausea, and pulsatile tinnitus (7). Initial options for IIH management include weight loss and carbonic anhydrase inhibitors such as acetazolamide (8). For patients with intractable diseases who fail or are intolerant of maximum medical therapy, surgical intervention is a potential option. The main surgical interventions available are optic nerve sheath fenestration (ONSF), cerebrospinal fluid (CSF) diversion with either lumboperitoneal or ventriculoperitoneal (VP) shunts, venus sinus stenting (VSS), and bariatric surgery (9). VSS is the most recently established procedure for IIH treatment and shows promising results in case series, but the details of the ophthalmic outcomes have not been well documented in large groups of patients. We retrospectively analyzed the ophthalmic and clinically important outcomes in a large series of consecutive patients who underwent VSS for IIH.

## Materials and Methods

### Patients

We performed a retrospective review of the medical records of patients who had undergone VSS at the Mayo Clinic, Rochester, Minnesota and Stanford Medical Center, Palo Alto, California, for the treatment of IIH between May 2015 and October 2021. This retrospective cohort study was approved by the Mayo Clinic and Stanford Medical Center institutional review boards. This study was conducted in accordance with the Health Insurance and Portability and Accountability Act (HIPAA) and adhered to the tenets of the Declaration of Helsinki.

The IIH diagnosis was based on the modified Dandy criteria, including the signs and symptoms of increased intracranial pressure, high intracranial pressure without ventricular enlargement or intracranial mass on imaging, and normal CSF constituents (10). Patients were considered for VSS if they met one or more of the following criteria: were refractory or intolerant to standard medical therapy; had failed prior surgical management; had persistent, progressive, or vision threatening papilledema, intractable headaches, and other symptoms of raised ICP despite maximal medical therapy. Patients who underwent cerebral venography received a venous sinus stent if the trans-stenotic pressure gradient was at least 8 mmHg (11–13). Patients were excluded from the study if they had VSS performed for non-IIH causes of raised ICP or if their post-VSS follow-up was less than one month. The electronic medical records of the patients were reviewed for baseline, intraprocedural, and postoperative clinical data.

### Demographic Data

Basic demographic data were collected for each patient, including age at VSS, most recent body mass index (BMI) before VSS, race, sex, and time between diagnosis and VSS.

### Pre-Venous Sinus Stenting Treatments for IIH

Attempted pre-VSS treatments for IIH were noted, including the use of standard medical treatments such as acetazolamide, topiramate, or furosemide, as well as prior interventional procedures such as ONSF, VP shunt placement, or bariatric surgery. Prior lumbar punctures were also noted, and the last known opening pressure before stenting was recorded.

### Clinical Signs & Symptoms and Ophthalmologic Assessment

**Figure 1:**
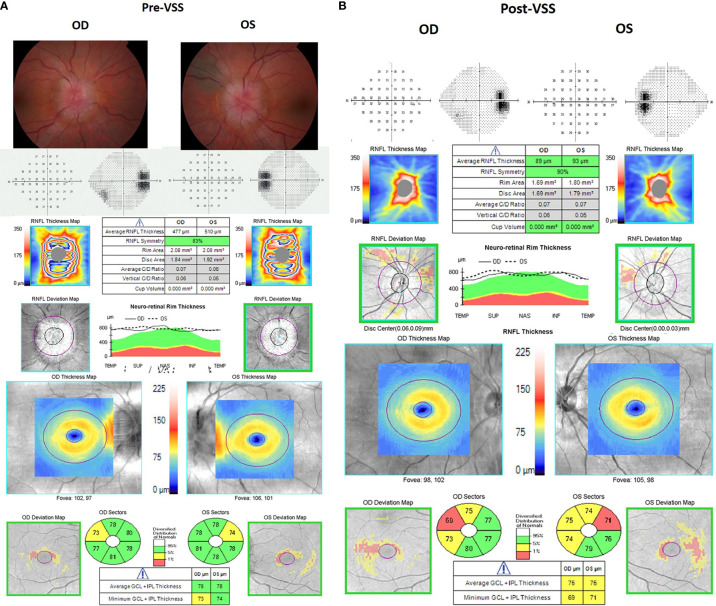
Illustrative fundus photos, visual fields, and optical coherence tomography pre- and post-venous sinus stenting (VSS) for a patient with idiopathic intracranial hypertension. Left panel **(A)** shows the ocular findings prior to VSS. The patient had bilateral grade 4 papilledema and significant enlargement of the blind spots on visual field testing with a trace nasal step in the right eye. Right panel **(B)** shows the ocular findings 3 months after VSS, showing resolution of the papilledema on OCT. Note that there were no fundus photos available for this patient post-VSS.

The presence of a headache or pulsatile tinnitus was noted before VSS and at follow up. Detailed ophthalmologic examination data were collected pre-VSS and at follow up, including best corrected visual acuity (BCVA), papilledema grade (Frisen scale), Humphrey visual field mean deviation (or manual kinetic Goldmann perimetry in cases of severe vision loss), and optical coherence tomography (OCT). To have a consistent post-operative comparison between patients, we chose to look at the 3-month post-VSS visit for the ophthalmic variables even though longer follow-up was often performed (**Figure 1**).

BCVA was measured using a Snellen chart and converted to LogMar for quantitative comparison. Humphrey visual field mean deviation (MD) was measured using a SITA standard 24-2 algorithm. Cirrus OCT was used to assess average peripapillary retinal nerve fiber layer (pRNFL) thickness and average ganglion cell inner plexiform (GC-IPL) thickness. The pRNFL thickness measurements were obtained using the optic disc cube 200 × 200 Cirrus protocol that covered the 6 × 6 mm^2^ area centered on the optic disc. The macular cube 512 × 128 Cirrus protocol centered on the fovea was used for the GC-IPL segmentation and measurements. The worse eye pre- and post-stenting was used for all comparisons. OCT images with segmentation errors, artifacts, and signal strength *<*6 were excluded to adhere to the APOSTEL and OSCAR-IB consensus criteria for OCT image quality (14, 15). For BCVA pre- and post-VSS comparisons, a change in visual acuity was defined as at least a 2-line change in the Snellen acuity chart in the worse eye. Patients were excluded from the visual acuity analysis if they had functional vision loss or did not have refraction or pinhole visual acuity recorded. For papilledema comparison, a change was defined as at least a 0.5 grade difference in the worse eye. For visual field mean deviation comparison, a change was defined as a minimum 2.0 dB difference from baseline in the worse eye. Unreliable visual fields (visual fields with a false-positive or false-negative rate of at least 10%) were excluded from the analysis. For pRNFL and GC-IPL thickness pre- and post-VSS comparisons, a change was defined as at least a 5 µm difference in the worse eye.

### Venus Sinus Stenting & Post-Procedural Therapy

Generally, procedures were performed under monitored anesthesia care with midazolam and fentanyl, with a propofol bolus administered at the time of stenting. Venous manometry and venography were performed at the same session as the stenting procedure, and no arteriography was performed. Generally, the femoral vein was cannulated using the modified Seldinger technique. Pressure measurements were obtained at the torcula and jugular vein, and if a pressure gradient greater than 8 mmHg was present, stenting was performed. Following stent placement, venography and venous manometry were performed. Procedures were performed under heparinization with 3,000–6,000 U of heparin administered at the beginning of the procedure. Patients were generally discharged home the day of the procedure on dual antiplatelet therapy (75 mg of clopidogrel daily and 325 mg of aspirin daily) for a minimum of 8 weeks (Stanford Medical Center) to 3 months (Mayo Clinic) post-VSS with follow-up CT venograms obtained to confirm stent patency. After 8 weeks or 3 months, clopidogrel was discontinued and low-dose aspirin (81 mg/daily) was recommended indefinitely. The trans-stenotic pressure gradient, the location of stent deployment, and any procedural complications were noted for each patient. If further interventional procedures were necessary post-VSS, such as repeat VSS, ONSF, VP shunt placement, or bariatric surgery, this was also noted.

### Data Analysis

Data analysis was performed using Microsoft Office Excel and Sigma Plot 14.5. Our primary outcome measures were change in BCVA, mean deviation, average pRNFL, and papilledema grade. Continuous variables were compared using the Student’s t-test and Mann–Whitney Rank Sum test, and categorical variables were compared using the chi-square test, with two-sided p-values and a significance level of 0.05. A Bonferroni correction was also performed to adjust the p-value for multiple (n = 5) variable comparisons with a significance level of 0.01.

## Results

Between May 2015 and October 2021, VSS was performed on 99 patients. Of these, 12 patients were excluded from our study because they did not have an IIH diagnosis, and 1 patient was excluded because their post-VSS follow-up was less than one month after their procedure, leaving 86 patients for analysis. Eight-two (95.3%) patients were female and 76 (88.4%) were of white race. The median age of the patients at the time of stenting was 33 (16–68) years, and the median BMI was 36.69 (22.30–62.00) kg/m^2^.

The median time between IIH diagnosis and VSS was 11.96 (0.21–419.14) months. Before VSS, 67/86 (77.9%) had papilledema, 85 (98.8%) patients reported headaches, and 68 (79.1%) reported pulsatile tinnitus (**Table 1**). For patients with 20/20 vision and no obvious visual field defects (n = 17), the clinical indication for VSS was the presence of debilitating headaches (n = 17) and pulsatile tinnitus (n = 15). Eighty-five (98.8%) patients had been on trial medical management for high ICP, with 55 (64.0%) patients having attempted 2 or more medications. Twelve (14.0%) patients had prior surgical procedures, with 1 patient having previously undergone both prior VP shunt placement and bariatric surgery. Eighty-five (98.8%) patients had prior LPs with the number of LPs ranging from 1 to greater than 100 in a patient who had bi-weekly therapeutic LPs over a 4-year time span. Only one patient did not have an LP before VSS; she had mild cerebellar tonsillar descent that was felt to be secondary to IIH rather than a congenital Chiari malformation. The median opening pressure of the last LP before VSS in our patient cohort was 310 (interquartile range, 264–370) mmH_2_O.

**Table 1:** Pre-VSS Baseline Clinical Characteristics and Procedure Details.

Clinical Characteristics (N = 86)	n (%)
Headache	85 (98.8%)
Pulsatile Tinnitus	68 (79.1%)
Prior Medications Attempted	85 (98.8%)
Acetazolamide	83 (96.5%)
Topiramate	45 (52.3%)
Furosemide/Equivalent	27 (31.4%)
More than 1 Pressure-Lowering Medication	55 (64.0%)
Prior Procedures Attempted	12 (14.0%)
Optic Nerve Sheath Fenestration	5 (5.8%)
Ventriculoperitoneal Shunt	6 (7.0%)
Bariatric Surgery	2 (2.3%)
Lumbar Puncture	85 (98.8%)
Number of Prior Lumbar Punctures	2 (1 to >100)*
Reason for Stenting	
Failed Max Medical Therapy	74 (86.0%)
Failed Max Medical and Prior Procedures	12 (14.0%)
Location of VSS Placement	
Right transverse sinus	68 (79.1%)
Left transverse sinus	17 (19.8%)
Left sigmoid sinus	1 (1.2%)
Procedure Complications	5 (5.8%)

*Estimates were used for patients who were unable to quantify the number of lumbar punctures.

The most common reason for VSS referral in our cohort was failed medical therapy, due to either lack of symptom relief at maximum therapeutic doses or intolerable side effects from the medications (74, 86.0%). Twelve (14.0%) patients had attempted both medical management and surgical procedures.

During VSS, the most common location for stent placement was the right transverse sinus (68, 79.1%), with the left transverse sinus being the second most common location (17, 19.8%). There was one patient who had a left sigmoid sinus stent placed because of procedural difficulty in placing a stent in either the right or left transverse sinus. The median pressure gradient across the stented segment was 18.5 (8–60) mmHg. There were 5 (5.8%) procedure complications noted, with 2 (2.3%) patients with cerebral hemorrhage (1 suffered cerebellar ataxia, 1 had transient headaches without neurologic deficits), 1 (1.2%) patient with femoral hematoma, 1 (1.2%) patient with transient post-operative seizures, and 1 (1.2%) patient reporting significant post-procedure pain.

Following VSS, 71 (83.5%) patients reported continued headaches, with 55/71 (77.5%) reporting improvement in headache quality, 12 (16.9%) reporting no change in headache quality, and 4 (5.6%) reporting worse headache quality (**Table 2**). Of the 68 patients that presented with pulsatile tinnitus, 40 (58.8%) reported complete resolution. 26 (32.1%) patients reported the presence of pulsatile tinnitus post-VSS, with 25 reporting persistent tinnitus and 1 reporting new tinnitus. Fifty-eight (67.4%) patients were still on a pressure-lowering medication for IIH following stenting, with 20 (23.3%) patients using 2 or more medications. There were 8 (9.3%) patients who had additional procedures following their initial stenting; 1 (1.2%) for improved but persistent papilledema, and 7 (8.1%) for persistent headaches. 1 (1.2%) patient had a VP shunt placed, while the remaining 7 (8.1%) had repeat VSS within 1.00 to 38.14 months after the initial VSS procedure. For the 7 patients who underwent repeat VSS, 2 (28.6%) underwent placement of stents at the same site for pressure gradients of 16 and 26 mmHg, while the remaining 5 (71.4%) underwent stent placement in the contralateral transverse sinus (4 of 5) and one in the sigmoid sinus, with demonstrated pre-stent gradients ranging from 11 to 60 mmHg. Sixteen (18.6%) patients had diagnostic lumbar punctures post-VSS, and the median LP opening pressure was 227 (170–310) mmH_2_O.

**Table 2:** Post-VSS Clinical Characteristics & Follow-up Procedures.

Clinical Characteristics	n/N (%)
Headache	71/85 (83.5%)
Improved	55/85 (77.5%)
Stable	12/85 (16.9%)
Worse	4/85 (5.6%)
Pulsatile Tinnitus	26/81 (32.1%)
Post-Procedure Medications	58/86 (67.4%)
Acetazolamide	51/86 (59.3%)
Topiramate	23/86 (26.7%)
Furosemide/Equivalent	8/86 (9.3%)
More than 1 Pressure-Lowering Medication	20/86 (23.3%)
Follow-up Procedures Post-VSS	8/86 (9.3%)
Ventriculoperitoneal Shunt	1/86 (1.2%)
Venous Sinus Stenting	7/86 (8.1%)

### Pre- and Post-VSS Ophthalmologic Findings

**Figure 2:**
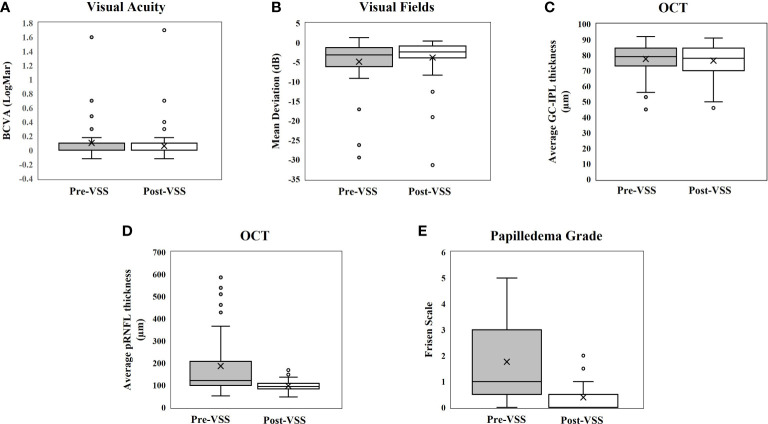
Box-whisker plots showing pre- and post-VSS comparisons for **(A)** Visual Acuity, **(B)** Visual Fields, **(C)** GC-IPL thickness, **(D)** pRNFL thickness, and **(E)** Papilledema Grade.

Of the 73 patients with pre- and post-VSS BCVA, the pre-VSS mean BCVA was 0.10 (−0.12–1.60), while the post-VSS mean was 0.06 (−0.12–1.7), *p* = 0.28 (**Table 3**, **Figure 2**, **Supplementary Figure 1**). Six (8.2%) showed an improvement in BCVA post-VSS of at least 2 lines, while no change in BCVA was observed in 67 (91.8%) patients, of whom 37 (50.7%) remained at their baseline of 20/20 vision post-VSS. No patient demonstrated worsening of BCVA. Among patients with a pre-VSS baseline BCVA of 20/30 or worse (n = 14), 5 (35.7%) improved, 0 (0.0%) worsened, and 9 (64.3%) were stable.

**Table 3:** Comparison of Pre- and Post-VSS Ophthalmologic Examination Findings.

Ophthalmologic Examination Finding, n	Pre-VSS Mean ± SD (Min–Max)	Post-VSS Mean ± SD (Min–Max)	*p*-value
Best Corrected Visual Acuity (LogMar), n = 73	0.10 ± 0.24 (−0.12–1.60)	0.06 ± 0.23 (−0.12–1.70)	*p* = 0.28
Visual Fields Mean Deviation (dB), n = 38	−4.87 ± 6.52 (−29.45–1.32)	−3.79 ± 5.87 (−31.4–0.44)	*p* = 0.44
OCT Average pRNFL Thickness (µm), n = 70	186.34 ± 136.37 (52.00–588.00)	96.86 ± 22.10 (47.00–168.00)	*p* <0.001
OCT Average GC-IPL thickness (µm), n = 57	77.56 ± 10.45 (45.00–92.00)	76.44 ± 10.87 (46.00–91.00)	*p* = 0.58
Frisen Papilledema Grade, n = 74	1.76 ± 1.48 (0–5)	0.39 ± 0.48 (0–2)	*p* <0.001

Of the 38 patients with pre- and post-VSS mean deviation, pre-VSS average mean deviation was −4.87 (−29.45–1.32) dB, while the post-VSS average was −3.79 (−31.4–0.44) dB, *p* = 0.44. Eleven (28.9%) showed improvement, 26 (68.4%) were unchanged, and there was 1 (2.6%) whose MD appeared to worsen post-VSS. For the one patient whose MD appeared to worsen, the patient’s MD went from −0.39 to −3.36 dB post VSS despite resolution of the papilledema and no further ganglion cell layer thinning. Among patients with a pre-VSS baseline mean deviation of at least −2 dB (n = 25), 11 (44.0%) improved, 14 (56.0%) were stable, and none worsened.

Of the 70 patients with pre- and post-VSS average pRNFL thickness, the pre-VSS mean pRNFL thickness was 186.34 (52.00–588.00) µm, while the post-VSS mean was 96.86 (47.00–168.00) µm, *p* <0.001. Fifty-four (77.1%) showed improvement, and 16 (22.9%) did not meet the preset criteria for change (≥5 µm change). Among patients with a pre-VSS baseline Frisen papilledema grade of ≥1 (n = 48), 45 (93.8%) showed at least a 5 µm decrease in pRNFL and 3 (6.3%) remained stable. No patients showed an increase in pRNFL after VSS.

Of the 57 patients with pre- and post-VSS average GC-IPL, the pre-VSS mean GC-IPL thickness was 76.56 (45.00–92.00) µm, while the post-VSS mean was 76.44 (46.00–91.00) µm, *p* = 0.58., 55 (96.5%) did not meet the criteria for change (≥5 µm change) and 2 (3.5%) appeared to worsen. In the two patients who had a thinner GC-IPL thickness at follow-up, there was severe papilledema and choroidal folds that may have caused artificial thickening of the baseline GC-IPL; visual function improved in both patients post-VSS.

Of the 74 patients with pre- and post-VSS fundoscopic examinations, the pre-VSS average papilledema grade was 1.76 (0–5), whereas the post-VSS average was 0.39 (0–2), *p* <0.001. Fifty-six (75.7%) showed improvement in papilledema post-VSS, and 18 (24.3%) remained unchanged at either trace or no papilledema pre- and post-VSS, with 12 (16.2%) having no papilledema. Among patients who had a pre-VSS Frisen papilledema grade of ≥1 (n = 48), all (100%) showed improvement after VSS. No patients had worsened after papilledema VSS.

Finally, pre- and post-VSS ophthalmologic findings were compared between sites (**Supplementary Tables 1, 2**), and there was no significant difference noted between the Mayo Clinic and Stanford Medical Center cohorts.

## Discussion

This large retrospective series highlights the generally excellent ophthalmic outcomes for patients with IIH who undergo venous sinus stenting. The most significant gains noted after VSS in our study were in papilledema and average pRNFL, which still achieved significance with Bonferroni correction. These findings conform to smaller prior studies. Touzé et al. demonstrated significant improvement in papilledema and RNFL thickness in as little as one month post-stenting in their 16 patient cohort (16). In their 2021 case series following 18 patients with IIH, Hendrix et al. showed a significant reduction in papilledema grade (*p* <0.001) and improvements in visual acuity (*p* = 0.012) and RNFL thickness (*p* <0.001), with less improvement in the mean deviation in visual fields (*p* = 0.119) after a median follow-up period of 8 months (17). Similarly, in the only prospective study available, Dinkin and Patsalides showed significant improvement in mean deviation, papilledema, and RNFL thickness among their 13-patient cohort following VSS, without any serious adverse events (16, 18).

To address the potential for post-VSS pRNFL and papilledema reduction to be interpreted as improvement as opposed to post-VSS atrophy, GC-IPL, a reliable indicator of atrophy (19), was also examined. Of the 57 patients with reliable pre- and post-VSS GC-IPL, there were only two patients with a significant reduction (at least a 5 µm difference) in GC-IPL, who both improved clinically. In addition, there was no new pallor detected post-VSS, and only one patient had a mild worsening in visual field mean deviation post-stenting on follow up despite observed clinical improvement. Therefore, the noted reductions in pRNFL and papilledema grade were attributed to lowered ICP from VSS as opposed to further atrophy, or new atrophy in a previously edematous optic nerve.

While improvements were also noted in a portion of our patients for BCVA and mean deviation, a smaller percentage met preset criteria for meaningful improvement than in most prior studies. For example, the 2019 Liu et al. study found 84.2% of patients reported significant improvement or recovery in their visual acuity (20). The difference is probably explained by baseline differences between the study population, as the baseline visual field mean deviation in the worse eye was −16.3 dB in the Liu et al. study compared to a baseline visual field mean deviation of only −4.90 dB in our study. The lower percentage of improvement in our study is likely due to a lack of pre-VSS baseline visual deficits in many of our patients, with 50.7% of the patients having 20/20 vision and 33.3% having normal visual fields before VSS, thus having no room for improvement. Among the patients presenting with a visual acuity worse than 20/30, 35.7% showed improvement in visual acuity, while 42.3% of the patients with a mean deviation less than −2 dB at baseline showed improvement post-VSS. As one of the few large studies of VSS for IIH, our study provides valuable evidence of the efficacy of VSS for visual and ophthalmic improvement in IIH.

Most of the stents (79.1%) were deployed in the right transverse sinus owing to the fact that stents are preferentially deployed in the dominant sinus, which is usually the right transverse sinus (21–23). The rate of major complications in our study was similar to the rates noted in the 2019 meta-analysis conducted by Nicholson et al. (1.9%; 95% CI 0.07-3.1%), further supporting the relatively good safety profile of VSS for IIH patients (24).

Symptomatically, there was improvement in most of our cohort, with 69 of the 85 patients (81.2%) reporting either complete resolution or improvement in the quality of their headaches and 55 of 81 (67.9%) reporting complete resolution of pulsatile tinnitus following VSS. While the rate of headache improvement in our cohort is similar to the 79.6% (95% CI: 73.3–85.9%) reported by the Nicholson et al. meta-analysis and other prior studies (24–26), the rate of pulsatile tinnitus resolution in our cohort was lower than the 90.3% (95% CI: 83.8–96.70%) reported in that meta-analysis. This discrepancy may be due to the fact that we did not capture patients whose pulsatile tinnitus improved because we only noted its presence or total absence post-VSS. In addition, in looking only at the 3-month post-VSS visit, it is possible that some patients had further improvement in pulsatile tinnitus later. Compared to prior studies reporting successful weaning of most of their patients off medication after stenting (16, 27), there were 58 (67.4%) patients in our cohort still requiring IIH management medication post-stenting. This, too, is likely due to the short follow-up period used for our analyses (4.0 months in our cohort versus 15.12 and 19.70 months in the Touzé and Zehri studies). We would expect more patients to be weaned off medications during longer follow-up. Of note, many patients require anti-headache medications even after normalization of intracranial pressure and resolution of papilledema, and this medication is often topiramate (tallied as an IIH management medication in this study).

Our rates for recurrent procedures at 8.1 and 1.2% were comparable to the 9% repeat endovascular procedure rate and 3% need for subsequent CSF diversion procedure reported in the Nicholson meta-analysis and lower than the 12.7 and 26% reported by Kahan et al. and Garner et al. (24, 28, 29). In their systematic review comparing various surgical treatment options for IIH, Kalyvas et al. concluded that VSS provides the best results for headache and visual outcomes with low failure and complication rates (9).

Our study has limitations, the greatest of which is the retrospective nature, which increases the risk of unknown confounders. Because it was a retrospective study, we were unable to obtain baseline and post-VSS data points on all patients at regular time intervals. The primary neuro-ophthalmologic outcomes were obtained at the 3-month visit post-stenting because we recommend post-VSS follow up with a complete neuro-ophthalmologic assessment and CTV 3 months after stenting to allow enough time for endothelialization of the stent and symptom stabilization, after which many patients continue regular follow-up with their local providers. This study cannot assess the long-term clinical outcome. Some patients were lost to follow-up shortly after stenting.

In conclusion, our study is one of the largest consecutive case series reporting on ophthalmic outcomes of VSS in patients with IIH. Our findings bolster smaller prior studies in demonstrating the overall efficacy and safety of VSS for patients with IIH, particularly in improvement in papilledema and pRNFL alongside overall clinical symptom improvement. Future prospective studies and randomized clinical trials of VSS are necessary to establish its long-term effect and place in IIH treatment guidelines.

## Data Availability Statement

The original contributions presented in the study are included in the article/**Supplementary Material**. Further inquiries can be directed to the corresponding author.

## Ethics Statement

The studies involving human participants were reviewed and approved by the Mayo Clinic institutional review board and Stanford Medical Center institutional review board. Written informed consent to participate in this study was provided by the participants.

## Author Contributions

JC, TX, KO, WB, and JCG contributed to conception and design of the study. KO organized the database. KO, TX, JC, WB, and HM performed data collection. JC and KO performed the statistical analysis. KO wrote the first draft of the manuscript. All authors listed have made a substantial, direct, and intellectual contribution to the work and approved it for publication.

## Conflict of Interest

Author HM has the following financial disclosures: NIH P30 026877 and an unrestricted grant from Research to Prevent Blindness.

The remaining authors declare that the research was conducted in the absence of any commercial or financial relationships that could be construed as a potential conflict of interest.

## Publisher’s Note

All claims expressed in this article are solely those of the authors and do not necessarily represent those of their affiliated organizations, or those of the publisher, the editors and the reviewers. Any product that may be evaluated in this article, or claim that may be made by its manufacturer, is not guaranteed or endorsed by the publisher.
