# Supplementary figures and images for: Improved Ophthalmic Outcomes Following Venous Sinus Stenting in Idiopathic Intracranial Hypertension
Source: Front Ophthalmol (Lausanne). 2022 Jun 30;2:910524. doi: 10.3389/fopht.2022.910524 (PMC11182255; doi:10.3389/fopht.2022.910524)

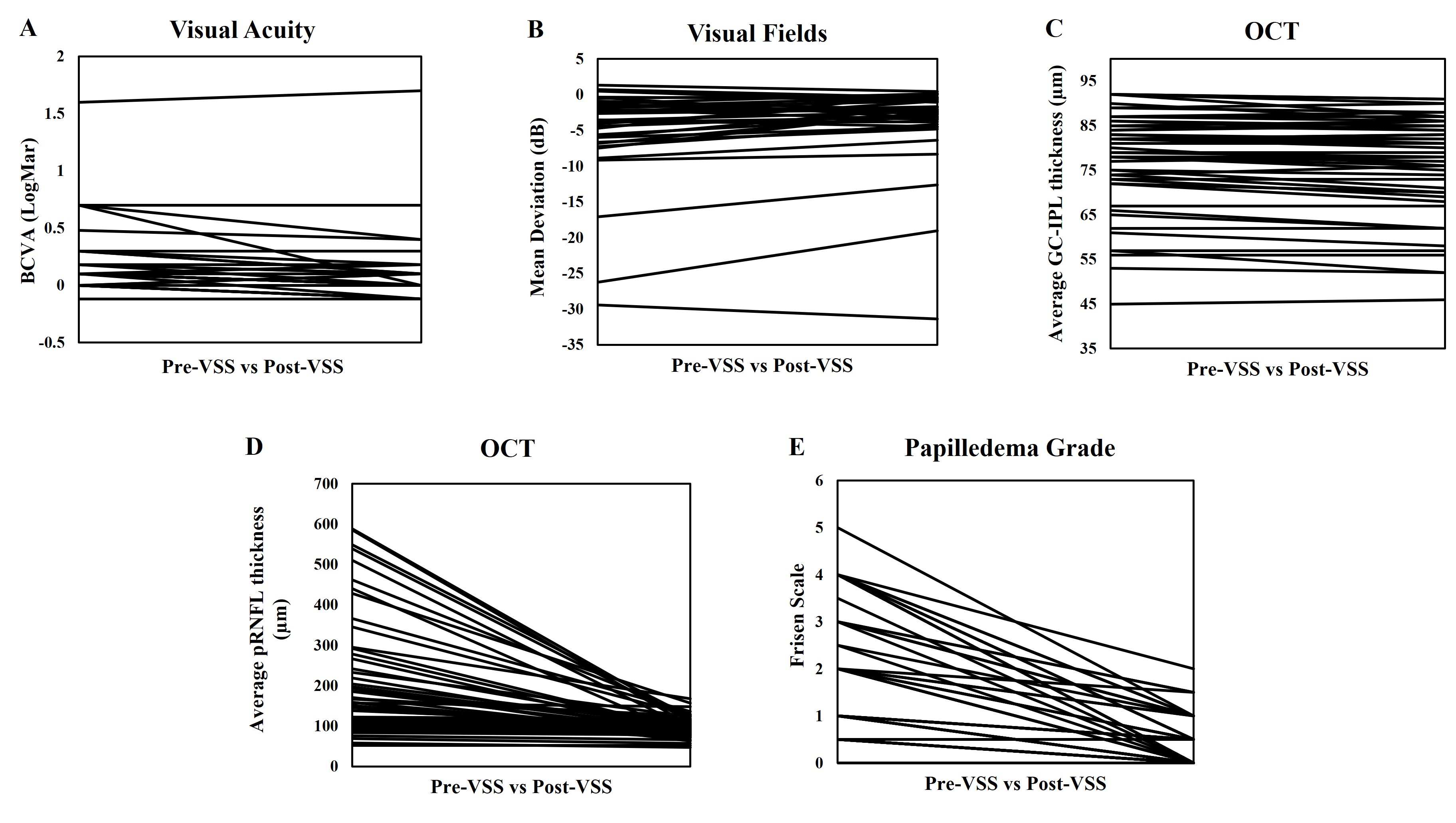

Supplement: Supplementary file 1 [file Image_1.jpg]
