# Supplementary material for: Improved Ophthalmic Outcomes Following Venous Sinus Stenting in Idiopathic Intracranial Hypertension
Source: Front Ophthalmol (Lausanne). 2022 Jun 30;2:910524. doi: 10.3389/fopht.2022.910524 (PMC11182255; doi:10.3389/fopht.2022.910524)
Supplement: Supplementary file 2 [file Table_1.docx]

## Supplementary Tables

**Supplementary Table 1.** Comparison of Pre-VSS Ophthalmologic Examination Findings by Site

| **Ophthalmologic Examination Finding** | **Pre-VSS Mayo Clinic**  **Mean ± SD (Min-Max)**  **n** | **Pre-VSS Stanford Medical Center**  **Mean ± SD (Min-Max)**  **n** |
| --- | --- | --- |
| Visual Acuity (LogMar) | 0.11 ± 0.25 (-0.12 – 1.60)  n=68 | 0.00 ± 0.00 (0.00 – 0.00)  n=5 |
| Visual Fields Mean Deviation (dB) | -4.87 ± 6.52 (-29.45 – 1.32)  n=38 | N/A |
| OCT Average pRNFL Thickness (µm) | 190.11 ± 140.52 (52.00 – 588.00)  n=65 | 137.40 ± 40.43 (104.00 – 204.00)  n=5 |
| OCT Average GC-IPL thickness (µm) | 76.94 ± 10.34 (45.00 – 92.00)  n=54 | 88.67 ± 5.77 (82.00 – 92.00)  n=3 |
| Frisen Papilledema Grade | 1.78 ± 1.51 (0 – 5)  n=69 | 1.50 ± 1.00 (0.0 – 2.5)  n=5 |

**Supplementary Table 2.** Comparison of Post-VSS Ophthalmologic Examination Findings by Site

| **Ophthalmologic Examination Finding** | **Post-VSS Mayo Clinic**  **Mean ± SD (Min-Max)**  **n** | **Post-VSS Stanford Medical Center**  **Mean ± SD (Min-Max)**  **n** |
| --- | --- | --- |
| Visual Acuity (LogMar) | 0.06 ± 0.24 (-0.12 – 1.70)  n=68 | 0.02 ± 0.04 (0.00 – 0.10)  n=5 |
| Visual Fields Mean Deviation (dB) | -3.79 ± 5.87 (-31.4 – 0.44)  n=38 | N/A |
| OCT Average pRNFL Thickness (µm) | 96.63 ± 22.78 (47.00 – 168.00)  n=65 | 99.80 ± 10.69 (88.00 – 114.00)  n=5 |
| OCT Average GC-IPL thickness (µm) | 75.80 ± 10.77 (46.00 – 90.00)  n=54 | 88.00 ± 5.20 (82.00 – 91.00)  n=3 |
| Frisen Papilledema Grade | 0.40 ± 0.48 (0 – 2)  n=69 | 0.20 ± 0.45 (0.0 – 1.0)  n=5 |

**Supplementary Figure 1.** Line charts showing individual pre- and post-VSS comparisons for **(A)** Visual Acuity **(B)** Visual Fields **(C)** GC-IPL thickness **(D)** pRNFL thickness and **(E)** Papilledema Grade
